# Application of urban growth boundary delineation based on a neural network approach and landscape metrics for Khulna City, Bangladesh

**DOI:** 10.1016/j.heliyon.2023.e16272

**Published:** 2023-05-16

**Authors:** Arpita Bakshi, Md. Esraz-Ul-Zannat

**Affiliations:** Department of Urban and Regional Planning, Khulna University of Engineering & Technology, Khulna-9203, Bangladesh

**Keywords:** Urban growth boundary, Artificial neural network, Landscape metrics, Boundary delineation

## Abstract

The rapid and unprecedented urban growth in Khulna, Bangladesh is making it difficult to implement measures to limit further expansion and define clear administrative boundaries, which is posing a significant threat to the environment and ecological sustainability. Using an Artificial Neural Network (ANN) based urban growth simulation model and landscape metrics, this study aims to evaluate the spatial extent and direction of urban growth and demarcate an Urban Growth Boundary (UGB) by examining the future contiguous expansion of the city for implementing effective land use provision. Utilizing data on biophysical, proximity, neighborhood, and market factors over the past twenty years, the neural network with Markov chain model allocates the land demand for buildup area by 2020 and 2030, concerning twelve explanatory variables. The simulated map of the urban area is further used by landscape metrics to quantify local-level urban patch information viz. landscape pattern, size, aggregation, etc. The compact patch characteristics are mostly found under the *Kotwali thana*, while, fragmented and unstructured patches are prevailing between urban–rural interfaces. Finally, there has around 95 km^2^ gap between the existing service provided by KCC and the future demand of Khulna city, creating an imbalance between the supply and demand of urban services. Hence, restricted urban growth would make government investment in service facilities cost-effective and enable planners and decision-makers to intend a feasible trade-off between future land demand and the protection of natural resources.

## 1 Introduction

About 67% of the world population will have expected to reside in urban and peri-urban areas by 2040 [1], primarily concentrated in Asia and Africa during 2018, enhancing regional importance over the years [2]. Likewise, the population of Bangladesh has increased rapidly from 71.48 million to 144.04 million between the years 1974 and 2011, respectively, moreover, 33% of the total population is estimated to reside in urban areas by 2021 [3]. Metropolitan cities, therefore, are enhancing their infrastructures and amenities through capital investment, which has led to disproportionate population growth and uneven urbanization, eventually impairing social and economic sustainability [[4], [5], [6], [7], [8]]

To ameliorate the effect of haphazard urban growth, several urban growth models, planning schemes, and policy interventions are adopted to resolve these issues [[9], [10], [11]] .And compact urban growth is deemed an effective urban management concept [[12], [13]] by comprising the UGB tool. UGB is a valuable regional planning tool concerning city compactness while protecting the natural resources of surrounding rural areas (Chettry & Surawar, 2021) [14,15]. Nevertheless, in Bangladesh, urban policies and strategies have not taken into account the concept and implementation of the UGB yet. Though it facilitates regulation and management of urban development by controlling the shape and direction of urban growth, excessive dependency on physical factors make reluctant the planning authorities consider it a viable urban management solution to assist them [16,17].

In recent decades, several models have been developed using GIS (Geographic Information System) and RS (Remote Sensing) techniques to demarcate UGB; for example, (1) hybrid approaches [18] (2) agent-based approaches [19] (3) SLR-UGB [17], (4) cellular automata-based model [[20], [21]], (5) machine learning model [[22], [23]]. Moreover, [24]prioritize ecological protection through dual-environmental evaluation and the future land-use simulation model to demarcate UGB along with landscape metrics. However, the published literature ignored directional changes in urban expansion over the years. Urban expansion intensity and land use gravity centers can precisely quantify the intensity and speed of urban growth in a certain period. Besides that, few studies took attempted to portray the shape and direction of future urban expansion by application of a simulation-based model, whereas landscape metrics are unable to simulate future urban growth. Additionally, most of the approaches ignore local level information (shape, configuration) of urban patches [[25], [26]], which is required to formulate a comprehensive plan to reduce urban sprawl [22,27].

The existing and newly generated urban patch information aids in classifying the traits of urban development, such as whether it is compact or fragmented as urban planners encounter difficulties regarding land-use fragmentation and urban sprawl. UGB delineation along with landscape metrics reduces these threats and protects natural resources as well. Our research applied the multilayer perceptron method to simulate future growth and landscape metrics for the shape and configuration of urban patches to demarcate a feasible UGB, which would enable planning authorities to ensure effective land-use provision and restrict haphazard urban expansion. Furthermore, it is evident from past studies that very little literature incorporates the size, shape, and direction of urban growth including landscape metrics for UGB delineation.

In practice, some fundamental regulatory frameworks have been addressed in the Five-Year Plans (FYP) to regulate future urban growth, however, Bangladesh is yet to have a sole regional or national plan to guide Khulna's urbanization in a planned manner [4,28]. As the third-largest metropolis (Khulna City Corporation, 1984), it will have faced population pressure on existing resources in the upcoming years, resulting in high land prices and urban service gap [29], hence, this study attempts to demarcate a UGB by encompassing demographic and market structure. In particular, this research aims to: (1) explore the existing spatiotemporal dynamics of land use land cover changes of the areas under the Jurisdiction of KDA (Khulna Development Authority). (2) simulate the future urban growth pattern using ANN and Markov chain and (3) delineate an Urban Growth Boundary using spatial analysis and landscape metrics.

## 2 Methodology

### 2.1 Study area selection

**Fig. 1:**
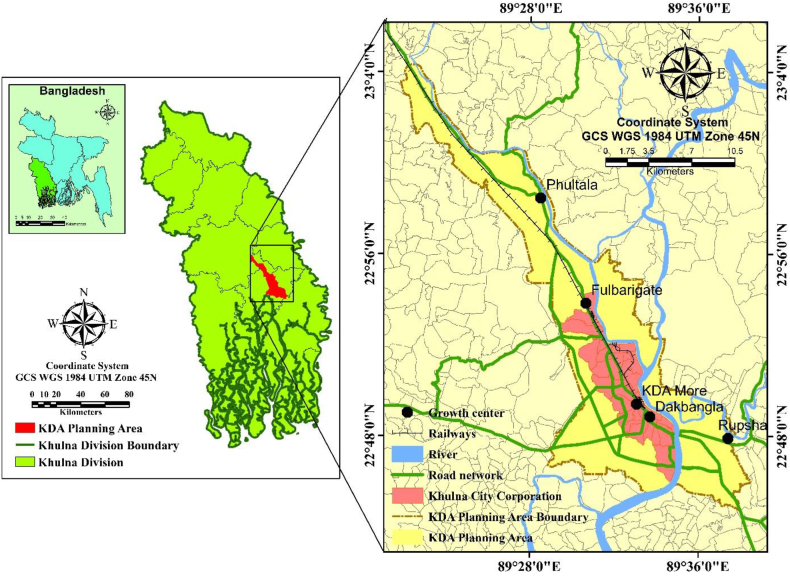
Study area map of Khulna and major road networks.

The study area covers a total of 1100 km^2^ area, including the KDA planning area (233.35 km^2^) except the Mongla Thana (Fig. 1). It also incorporates Khulna City Corporation (KCC) (41.34 km^2^). KDA planning area is located along with Bhairab-Rupsha rivers between the latitude and longitude of 23°4′19″ to 22° 45′ 00″ N and 89°22′30″ to 89°37′34″ E and is surrounded in the south by Jessore and Narail, west of Bagerhat, east of Satkhira district, and north of the Bay of Bengal. Moreover, the natural levee on the sides of those rivers is the most suitable location for human settlement growing up on the west of the Bhairab-Rupsha River, extending to the northwest (Urban Strategy, Khulna city, 2000). The population density of KCC was 16268 per km^2^, with a 22.11% population growth rate between 1991 and 2011 [30].

The linear shape along with the Bhairab-Rupsha rivers has played a vital role in shaping the city as a linear pattern extending from southeast to northwest [29] . Besides that, due to the establishment of the Khulna-Jessore road, it has started to extend towards the Khulna-Jessore road. After the development of the Dhaka-Khulna corridor under the Southwest Road Network Development Project, the strategic importance and connection between Dhaka and the towns of Mongla, Khulna, Jessore, and Benapole has created opportunities for greater regional cooperation with neighboring countries and stimulated economic growth in the country's relatively neglected southwestern area. Furthermore, important railways and roads link boost trade and commerce and revitalize economic transactions [31].

Khulna DAP (2018–2023) has been approved just in 2018, however, the development authorities fail to accommodate the Structure Plan, Master Plan for Khulna City on time. Along with that, different city boundaries defined by different authorities have made the planning process complicated, creating confusion and delay. Those issues drive the concerned authorities to exclude or include areas inside or outside master plan zones while proposing plans [4].

### 2.2 Spatial database preparation

Three types of spatial datasets were used including vector-based data, remote sensing imageries, and statistical data. USGS Earth Explorer was used to extract Landsat satellite images for the years 2000–2020 at 5-year intervals (cloud cover <10%). After obtaining the images, preprocessing was carried out in the QGIS interface by applying a Semi-Automatic Classification Plugin (SCP), comprising Dark Object Substruction (DOS) method to correct the images atmospherically and radiometrically as well as other factors that might have an impact on the accuracy of the images. Afterward, data preparation took place to organize the data for further analysis by selecting suitable bands of required spatial resolution. Lastly, data processing was operated by applying algorithms and techniques to extract relevant information. The dataset containing surface reflectance was derived from the different bands of Landsat 4–5 TM and Landsat 8 OLI (Table 1), with zone 45N and datum WGS-84, to show the temporal and spatial variation of land use land cover over the years. Moreover, Digital Elevation Model (DEM) data was used to portray the existing topography and slope of the area, extracted from USGS. The administrative boundary map of KDA was used to define the area of interest, whereas maps of existing roads, railways, and rivers were generated by data extracted from the ‘one street map’. In addition, population data of the study area was extracted from ‘District Statistics 2011. Khulna[30] whereas land price data for 2013 were collected from KDA. The population and land price data were extrapolated for 2020 using the linear extrapolation method.

**Table 1:** Description of landsat images downloaded from USGS.

Date Acquired	Scene ID	Sensor	Cloud Cover	Path/row
2000/04/08	LT05_138044_20000517	Landsat 4–5 TM C1 Level-1	<10%	138/44
2010/06/19	LT05_138044_20100411	Landsat 4–5 TM C1 Level-1	<10%	138/44
2020/06/31	LC08_138044_20200406	Landsat 8 OLI/TIRS C1 Level-1	<10%	138/44

### 2.3 Identification of explanatory factors

Driving factors influence the landscape pattern including spatial, political, socioeconomic, and technological forces [32,33]. In highly urbanized areas, the changing pattern of land use is significantly affected by the factors addressed in existing literature depending upon the availability of data, features, and nature of the study area (Table 2).

**Table 2:** Factors selected to analyze future urban growth in KDA planning area under Khulna.

No.	Name	Purpose
(a)	Topography	Assessing the elevation of land
(b)	Slope	Measuring the percentage of change in elevation over certain distance of land
(c)	Distance from nearest buildup area	Assessing distance to old buildup area as it encourages to accelerate new buildup area around it
(d)	Distance from city center	Assessing the proximity to city centers that define the high-density built-up areas
(e)	Distance from major roads	Assessing the proximity to major roads fragmenting natural landscape pattern
(f)	Distance from minor roads	Assessing the proximity to minor roads reflect the buildup areas around it
(g)	Distance from railways	Assessing the proximity to railways
(h)	Buildup density	Assessing the concentration of buildup area account for the vicinity of existing buildup area
(i)	Cropland density	Assessing the concentration of cropland area account for the vicinity of existing buildup area
(j)	Normalized land value	Forecasting the land value for future urban growth
(k)	Population density	Forecasting the population density for future urban growth
(l)	Constraints	Dealing with the location of water bodies restrict from future urban Land use

**Fig. 2:**
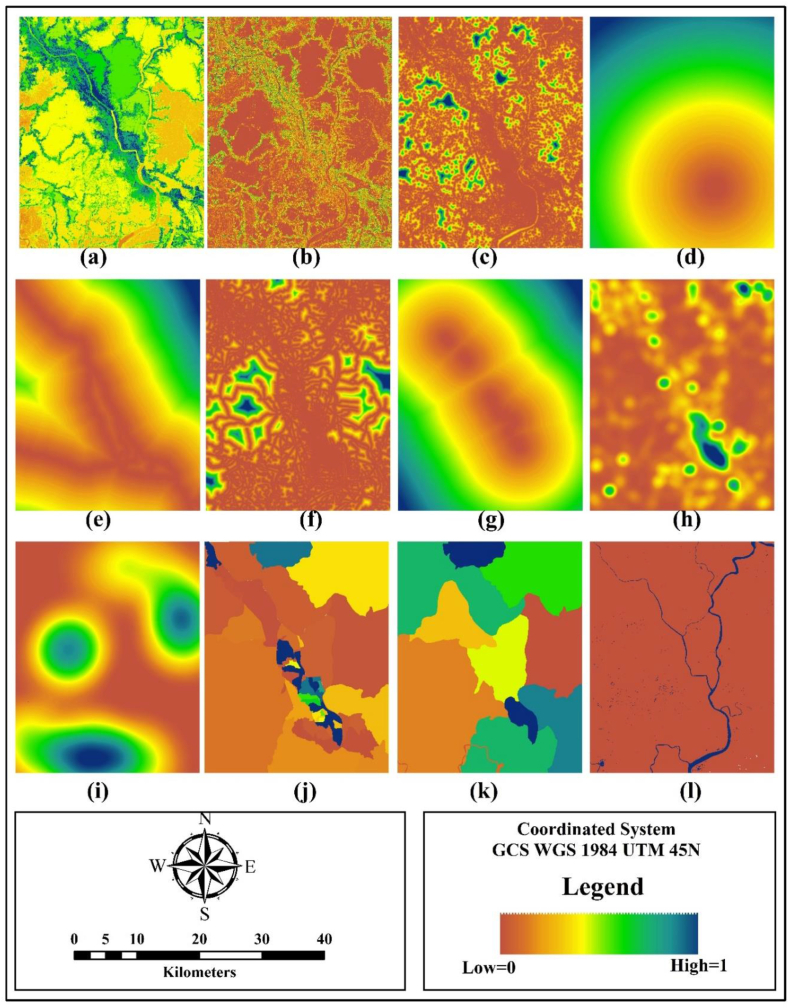
Visualization of selected factors for simulating urban growth.

Explanatory variables were divided into four broad classes viz. biophysical factor, proximity factor, neighborhood factor, and market factor (Fig. 2), with a processing extent of 991 × 1235. Biophysical factor: The surface physiography of Khulna has characterized by various geomorphic units with different land levels [[29], [34], [35], [36], [37]] . Topography gradually decreases sharply to the east and west direction from the main Khulna city. Topography and slope were used as biophysical factors influencing the pattern of urban growth. Proximity factor: The linear shape of Khulna city along with the Bhiarab-Rupsha rivers are exceeding the southeast to northwest [[29], [38]] . Most of the urban growth was formed in the vicinity of road networks and played a vital role to shape the city into a linear one. Additionally, railways, roads, and waterways transport network maintained a proximity towards south to the north direction (Detailed Area Plan, Khulna city, 2000). Proximity factors were generated using Euclidean distance (e.g. distance to the city core, major roads, minor roads, railways, nearest buildup area) in GEE (Google Earth Engine) interface.

The concentration of buildup area and cropland area are some dominant factors accelerating urban growth named neighborhood factors [39,40]. Neighborhood factors: it was used to incorporate the density of buildup area and agricultural land using kernel density (Structure Plan, Khulna city, 2002). Socio-economic factors: Growth of population and land value are the two most interrelated aspects of the urban growth of Khulna. Land value is a vital determining factor for the physical expansion of a city in a particular direction [22]. A higher value of land has commercial importance which is the reason for the growing affluent residential area under the KDA planning area (Structure Plan, Khulna city, 2002). Socioeconomic factors included the population census data and land value dynamics that were collected from the census data of Bangladesh. Constraints: conservation of existing resources for future use is necessary for tremendous urban growth as well as ensuring ecological viability. A Binary layer projecting the major rivers and water bodies under the study area was created.

### 2.4 Model development and optimization

**Fig. 3:**
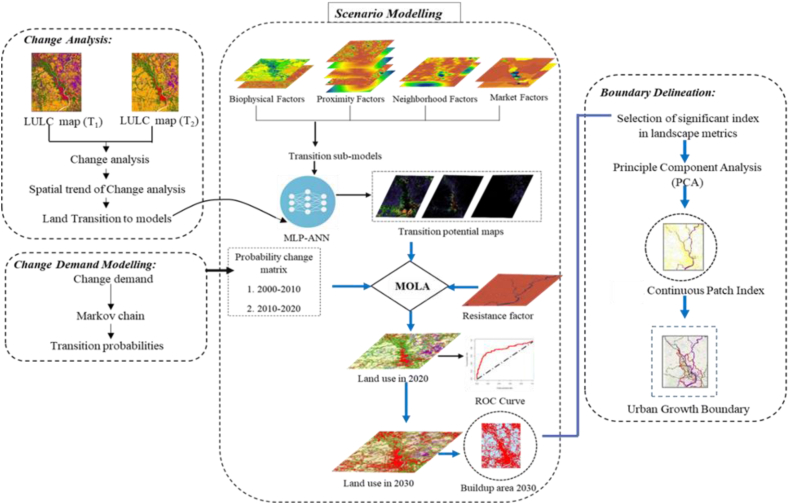
A schematic framework of model development and delineation of growth boundary.

The flow chart of the overall methodology is followed in (Fig. 3). The study applied a neural network-based simulation model to produce maps for delineating UGB. This model generated the future urban spatial allocation and direction for demarcating UGB.

#### 2.4.1 Land use data processing and accuracy assessment

The maximum likelihood supervised classification method was applied to generate LULC maps from 2000 to 2020 at 10-year intervals. Five broad classes were identified to be mapped: Water body, Buildup area, Bare land, Marshland & Open Agricultural Land (MOAL), and vegetation (Table 3). The number of training samples was fixed at 200 for each feature class.

**Table 3:** Description of land cover types.

Land Cover Type	Description
1. Water body	Lakes, Ponds, Cannels, River, Permanent open water.
2. Buildup Area	Residential, Commercial, Industrial Infrastructure, Rural settlements, Neighborhoods, Infrastructure for transportation.
3. Bare Land	Bare Land, Open space, Exposed soil.
4. Marshland and Open Agricultural Land (MOAL)	Low-lying area, All types of wetlands, All types of cultivated land (urban agriculture, crop field), Fish farm.
5. Vegetation	Trees, shrub, inner-city recreational areas, parks and playgrounds, grassland, vegetable lands, Urban forest, Mixed forest.

The accuracy of the land use land cover maps was assessed by two methods. Firstly, almost 100 reference samples were taken for each feature class from Google Earth imagery for the years 2000, 2010, and 2020. After that, the accuracy of the map of 2000, 2010, and 2020 was calculated by conducting a confusion matrix with commission error, omission error, overall accuracy, and kappa coefficient [41]. Moreover, the classified image of 2000 was also validated by old policy documents of Khulna like structure plan, master plan, and detailed area plan. For better accuracy, ground truth data for 2010 and 2020 were taken from previous research data and physical surveys as 50 per land use class.

#### 2.4.2 MLP (multilayer perceptron) neural network

MLP method is widely used in various aspects of urban planning to simulate future urban expansion [17,22,42]. The multi-layer perceptions are interconnected where neurons of each layer are linked with the neurons of neighboring layers. The concept of MLP can be expressed by the following basic equation:

(1) y=f(W2*f(W1*x+b1)+b2)

where *x* is defined as the input vector. Here, W represents the weight matrix, where W1 can link the input layer with the hidden layer and W2 can connect the hidden layer with the output layer. Additionally, b1 and b2 are the bias vector for the hidden layer and output layer, respectively. f is an activation function that represents the nonlinearity of the output layer (e.g. tanh, sigmoid, ReLU, etc.). Finally, y is the output vector [43].

**Fig. 4:**
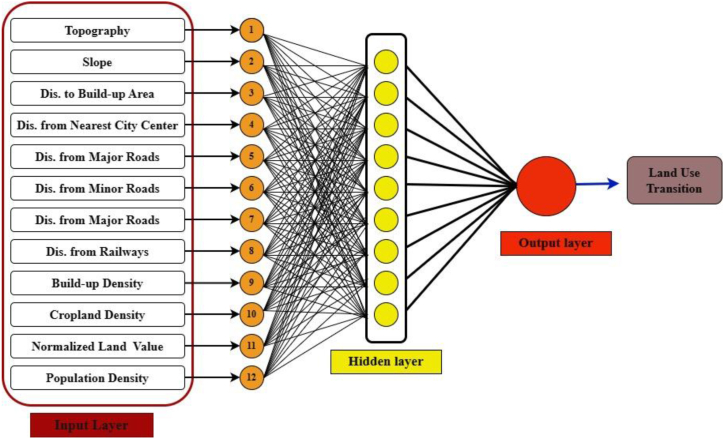
A framework for ANN architecture to predict land use transitions.

The simulation model makes use of the MLP network of Land Change Modeler (LMC) (https://clarklabs.org/terrset/land-change-modeler/). TerrSet software was used to predict the urban growth of 2020 by using the explanatory variables as inputs in the LCM interface (Fig. 4). The selection of the explanatory variable depends on the test of Cramer's V [44]. It can measure the explanatory power of each driver in creating urban growth modeling. The value of each driving factor as 0.15 or higher than 0.15 was considered useful for explaining urban growth whereas 0.4 or above has good explanatory power [45].

MLP can model multiple transitions into one sub-model by creating input, hidden, and output layers. The hidden neurons enable the analysis of complex scenarios, where there have 9 hidden neurons with land use transition maps. Moreover, three transition sub-models (e.g. bare land to buildup area, vegetation to buildup area, Marsh, and open agricultural land to buildup area) were obtained between the transition period of 2000 and 2010. For each transition period, a 2500 sample size was taken as training and testing samples with 10000 iterations and 0.01 learning rate (Table 4). The number of iterations was selected by the trial and error method. An acceptable accuracy rate is 80%, and it can be evident that the MLP has learned subsequently from the input data [46] In our study, 88.65% accuracy rate was recorded.

**Table 4:** Training parameters of MLP-ANN.

Network parameters	Value
Start learning rate	0.01
Momentum factor	0.5
Sigmoid constant a	1.0
Hidden layer nodes	9
RMS	0.01
Iterations	10,000

#### 2.4.3 Land allocation and change demand modelling

Change prediction in the lens of the Markov Chain Model generated a transition probability matrix to predict the probability of change from one land use to another using TerrSet software. The model quantified the future land demands for each land use class for the intended year of prediction, which were subsequently transformed into transition matrices. The formula of MOLA can be applied as follows in the TerrSet LCM:

(2) minimizef(x)=(f1(x),f2(x),...,fm(x))

Subject to g(x) ≤ 0, h(x) = 0, lb ≤ x ≤ ub. Where x can be portrayed as a raster or vector layer which represents the land allocation of different land use classes and f(x) is defined as a vector of the objective function m. Here, g(x) addresses the constraints of land use activities in the form of a set of raster or vector layers, whereas h(x) represents the requirements that must be accomplished. lb is the lower bound and ub is the upper bound on the land use activities.

Due to the extinction of waterbodies and shrinkage of rivers over the years, this study aimed to allocate the land demand for transition rate, which is considered as a conservation restriction policy. Later, two transition probabilities (2000–2010 & 2010–2020), one constraint, and twelve explanatory factors were accounted for predicting the land use map of 2020 and 2030. The LULC classes were eventually predicted in the TerrSet environment based on these scenarios. Furthermore, TerrSet has build-in Multi-Objective Land Allocation (MOLA) function, which allocated the land demand for buildup areas by 2020 and 2030. LCM went through the transitions and generated a list of host classes and claimant classes. Later, MOLA allocated land considering transition potential maps for each claimant of a host class [47] The internal module of MOLA named CHGALLOC resolves the conflicts regarding the allocation of land for the Claimant's class. The predicted result was then generated by overlaying the reallocation result from each host class [42]. Thereafter, two transition index maps were obtained where first one (2000–2010) and Second one (2010–2020) was created based on the driving variables of 2000 and 2010, respectively.

#### 2.4.4 Model validation

Model validation mainly refers to the comparison between simulated and actual urban growth dynamics. To validate the model, ROC (Receiver Operating Curve) curve, a binary classifier was used to indicate model performance, where AUC (Area under the Curve) summarized the overall accuracy of the test. The value of AUC was considered between 0 to 1 where 0.50 depicts no discrimination, 0.7 to 0.8 as acceptable, 0.8 to 0.9 as excellent and more than 0.9 as outstanding predictions [48].

### 2.5 Directional change of urban growth

#### 2.5.1 Urban expansion intensity index (UEII)

Directional assessment of urban expansion is one of the most common techniques for measuring the intensity and speed of urbanization in different directions. Differentiating the expansion rate in distinct sectors is made easier by the visual input of diverse urban growth in different directions [49]. 2 km multiple-ring buffer in 16 gradient directions was created surrounding the city center for micro-scale observation.

Spatial expansion of urban areas was calculated by UEII to identify the preferences of urban growth and evaluate the speed and intensity of changes of LULC over the years as they are the vital factors to quantify directional urban increase [50]. The formula for calculating UEII is shown in Eq. (1) [51].

(3) $${UEII}_{it}=\left[\frac{{ULA}_{i,b}-{ULA}_{I,A}}{t}\right]/{TLA}_{i}\times 100$$

Here, ${UEII}_{it}$ = Annual Average Urban Expansion Intensity of $i^{th}$ Zone in Time Period t. ${ULA}_{i,a}$ = Quantity of Buildup Area at Time Period a in $i^{th}$ Zone. ${ULA}_{i,b}$ = Quantity of Buildup Area at Time Period b in $i^{th}$ Zone. ${TLA}_{i}$ = Total Area of $i^{th}$ Zone.

#### 2.5.2 Land use gravity center

The center of gravity of buildup land use was used to represent the change of spatial distribution for certain LULC classes [52]. Urban gravity center is a useful index which indicates the correlation between urban growth and policy making. Spatial statistics were adopted to measure the coordinates of the gravity center of the buildup area over the years. Eq. (2) depicts the calculation formula of gravity center as below:

(4) $$X_{m}=\frac{\sum_{i+1}^{n}\left(A_{mi}\times X_{mi}\right)}{A_{m}}$$

(5) $$Y_{m}=\frac{\sum_{i+1}^{n}\left(A_{mi}\times Y_{mi}\right)}{A_{m}}$$

$X_{m}$ = Gravity Center of Buildup Area's Value of X-Coordinate in the Year *m*. $Y_{m}$ = Gravity Center of Buildup Area's Value of Y-Coordinate in the Year *m*. $A_{mi}$ = Area of Buildup Land use of polygon i in the year *m*. $X_{mi}$ *=* Average of X-Coordinate. $A_{m}$ = Total Area of Buildup Land use in the year *m.*

### 2.6 Delineation of Urban Growth Boundary

The third objective was accomplished by the boundary delineation of the urban growth area. In the present study, some selected landscape metrics were accounted for to compute the UGB which facilitates the future characterization of pattern, shape, and aggregation by using Fragstat 4.2. In addition, landscape metrics were used over time to explain and compute the spatial characteristics of patches, the area of Land use cover, and the entire land cover. It is useful to track, calculate and evaluate changes in Land use land cover to detect the changes due to urban sprawl and its structure [53].

#### 2.6.1 Selection of landscape metrics

Landscape metrics have a diverse index that may or may not be significant to our study area. Therefore, the variables were selected according to earlier work reported in urban studies [[22], [32], [54]]. In this research, we selected eight landscape matrices for simulated buildup areas of 2030; PLAND (Percentage of Landscape), LSI (Landscape Shape Index), LPI (Largest Patch Index), FRAC_AM (Fractal Dimension Index), COHESION (Patch Cohesion Index), AI (Aggregation Index), PD (Patch Density), SPLIT (Splitting Index). FRAGSTAT 4.2 was applied to calculate the relevant indexes to represent the shape and pattern of urban patches (Table 5), using 4-cell neighborhood rules. The window size was selected as 500m*500m among 100m*100m, 300m*300m, and 800m*800m showing the best output.

**Table 5:** Descriptions of landscape metrics used for delineating UGB [[55], [56]]

Aspect of pattern	Matric name	Symbol	Acronym unit	Description	Range
Area and Edge	Percentage of Landscape	PLAND	%	The aggregated area of landscape	0–100
	Landscape Shape Index	LSI	Dimensionless	It is the normalized ratio of edge of an area where the total length of edge is compared with a standard shape of landscape.	0–1
	Largest Patch Index	LPI	%	Ratio between the corresponding patch type's largest patch and the total landscape area.	0–100
Shape	Fractal Dimension Index	FRAC_MN	Dimensionless	A normalized shape index measures log transformed perimeter and area	1 ≤ to ≤2
	Patch Cohesion Index	COHESION	%	It is proportional to the area-weighted mean perimeter-area ratio which is divided by area weighted mean patch shape index	0–100
Aggregation	Aggregation Index	AI	%	It is the tendency of each patch type to aggregate spatially. It defines by the ratio of the observed and maximum likelihood of possible adjacencies for each patch type.	0–100
Subdivision	Patch Density	PD	Number of patches per 100 ha	The number of patches per unit area	PD ≥ 1, no limit
	Splitting Index	SPLIT	Dimensionless	The number of equal sized patches by subdividing the total landscape based on effective mesh size	SPLIT≥1, no limit
Urban Continuous Patch Index		UCPI	None	It is the single largest continuous patch. In this the method of dimension reduction (PCA) is used to capture the form, pattern, region-edge, aggregation, etc.	UCPI≥ 0, no limit

#### 2.6.2 Urban continuous patch index (UCPI)

UCPI acted as a spatial metric to represent the information about urban patches and was measured by the ratio of the total area of contiguous/continuous patches of buildup area and the total study area. The maximum value of UCPI defines the higher connectivity between the urban lands and the lower value indicates less continuity. Higher connectivity indicates how well the urban areas are linked to each other to mobilize goods and service facilities. In the present study, selected landscape metrics helped to compute the UCPI for better representation of shape, cohesion, aggregation, and the fraction of urban patches. After the selection of metrics, the correlation between the variables was analyzed. the highly significant correlation (>0.75) between the independent variables poses a challenge in research called the multicollinearity problem [[57], [58]]. Principle Component Analysis (PCA) is a solution to overcome this problem to reveal the landscape structure properly [[58], [59]] by using OriginPro2021. Furthermore, PCA was used to aggregate those metrics into UCPI for 2030 which can provide a more accurate depiction of the shape, edge, and aggregation of urban patches.

##### 2.6.2.1 UGB delineation

UGB quantifies a hard-administrative boundary for ensuring feasible land allocation as well as protecting the environment, however, having some uncertainties. Here, we quantified hard boundary of contiguous urban area for the year 2030, which was extracted from UCPI by canny edge detector in Python using OpenCV library. As UCPI has some noises in the urban cells as well as fragmentation, canny edge detector processed the image to clarify the growth boundary. Afterwards, the whole image was converted into a polygon and intersected with the boundary of the KDA planning area.

## 3 Results

### 3.1 Land use classification and change analysis

**Fig. 5:**
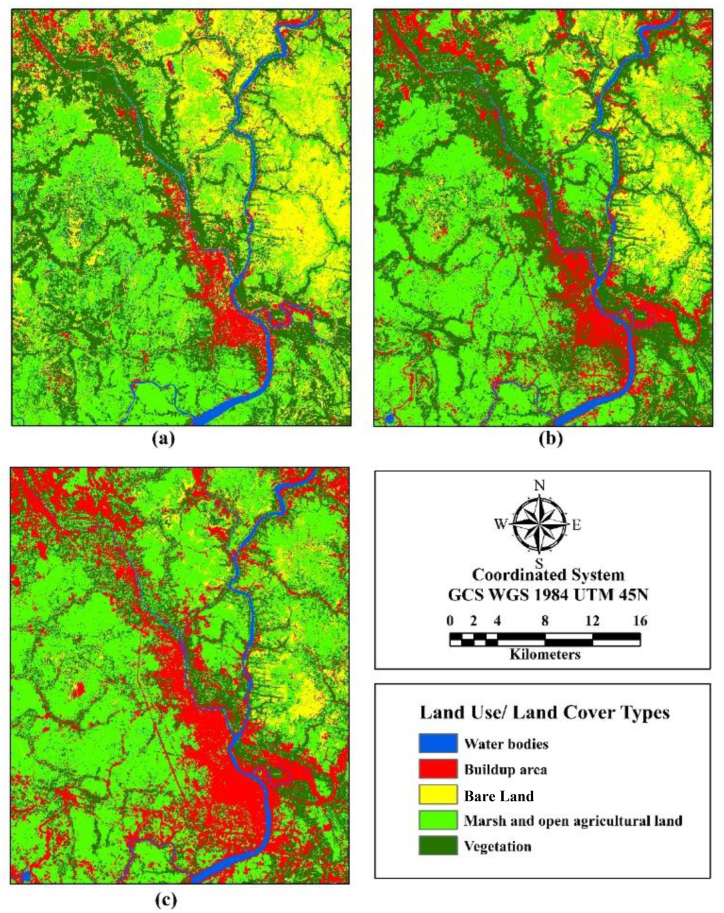
Classified image of different LULC classes for (a) 2000, (b) 2010, (c) 2020.

The land use change of the study area was represented by the LULC map of the years 2000, 2010 and 2020 (Fig. 5(a–c)). Moreover, the value of the Kappa coefficient for the years 2000, 2010, and 2020 classified images were calculated as 0.81, 0.93 and 0.90 respectively, which portray a good accuracy. During 2000, the buildup area comprised of 67km^2^, which was 6% of the total study area. In addition, vegetation, MOAL covered 357 km^2^ and 456 km^2^ areas, respectively, whereas water bodies and bare land occupied 39 km^2^ and 179 km^2^ areas, respectively (Table 6). It also shows that over the 2010 to 2020 time period, buildup areas increased 110 km^2^–194 km^2^ with a rate of 0.77 km^2^ per year. Nevertheless, vegetation and bare land disappeared at the rate of 0.11 km^2^ and 0.41 km^2^ per year, respectively for the same period. Moreover, 0.14 km^2^ of water bodies was recorded to reduce per year between 2010 and 2020.

**Table 6:** The area of each Land use land cover class of 2000, 2010, 2020.

Land use land cover types	Area (km^2^)		
	2000	2010	2020
1. Water bodies	39.51	32.11	27.68
2. Buildup area	67.63	110.08	194.32
3. Bare land	179.22	77.15	45.50
4. Marshland and open agricultural land (MOAL)	456.22	618.42	596.42
5. Vegetation	357.42	263.28	238.03

**Fig. 6:**
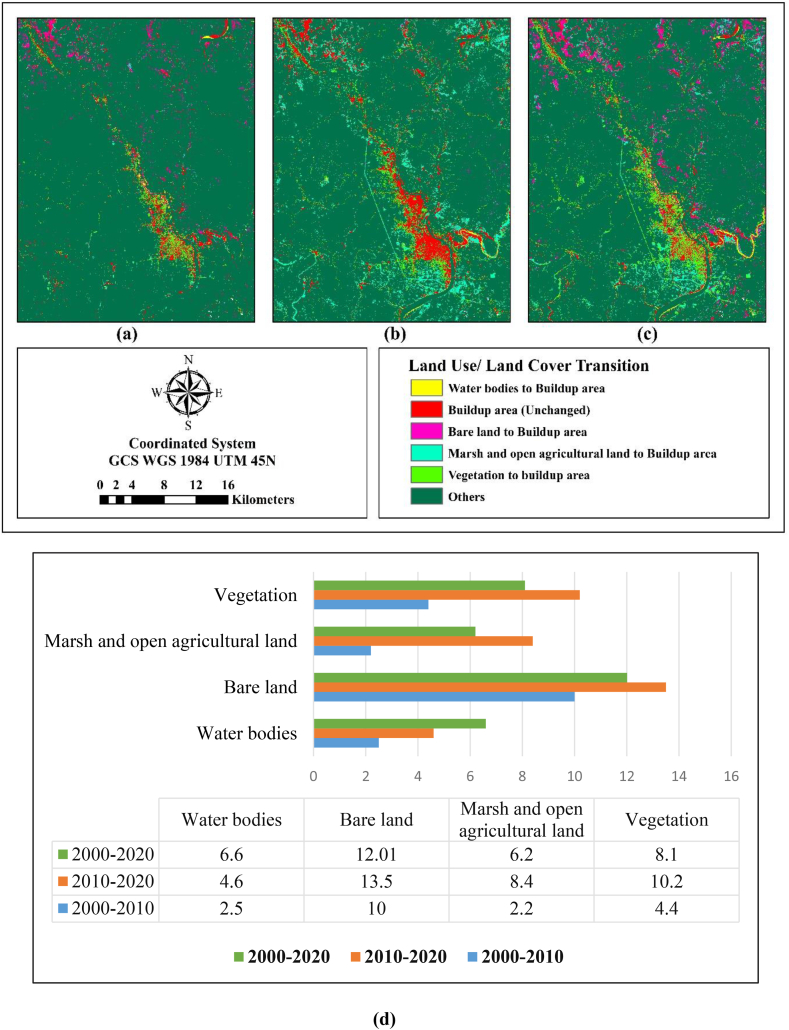
Transition map from other class to buildup area (a) 2000–2010 (b) 2010–2020.

Fig. 6(a–c) represents the transition map for 2000–2010, 2010–2020 and 2000–2020. From 2000 to 2010, the contribution of bare land to convert into buildup area was 10% in comparison to 13% in 2010–2020 which is in total 17% during 2000–2020. Likewise, MOAL accounted to 2% and 8%, respectively, during the time frame of 2000–2010 and 2010–2020 (Fig. 6(d)).

### 3.2 Analysis of driving factors

The values of Cramer's V demonstrated the level of association with 11 driving factors and buildup area in the time frame of 2000–2010 and 2010–2020. The usefulness of the model implies the overall values higher than 0.15 for buildup areas [42]. Buildup area proximity had the highest association with urban growth in both the period of 2000–2010 and 2010–2020. Moreover, distance to major roads, C.B.D, buildup density, population density, and land price were highly associated between 2010-2020 (Table 7).

**Table 7:** Explanatory variables and associated Cramer's V coefficient.

Explanatory variables	2000–2010		2010–2020	
	Overall Cramer's Value	Build up Area	Overall Cramer's Value	Build up Area
Nearest buildup area	0.31	0.52	0.52	0.82
C.B. D	0.21	0.39	0.21	0.41
Major roads	0.25	0.38	0.20	0.47
Minor roads	0.21	0.26	0.18	0.24
Railways	0.20	0.25	0.16	0.24
Buildup density	0.26	0.40	0.27	0.45
Cropland density	0.16	0.18	0.15	0.18
Population density	0.20	0.45	0.18	0.43
Land price	0.26	0.33	0.23	0.38
Elevation	0.32	0.24	0.28	0.20
Slope	0.26	0.25	0.19	0.18

**Cramer's V of 0.15 or higher is useful while those with values of 0.4 or higher are good.

### 3.3 Future urban growth scenario

Changing pattern of urban growth and significant explanatory variables conducted a transition sub-model via MLP neural network to portray the future transition potential of each land class converting into a buildup area. This also includes Markov Chian to produce metrics of Land use conversion.

#### 3.3.1 Transition potential modelling and land demand calculation

**Fig. 7:**
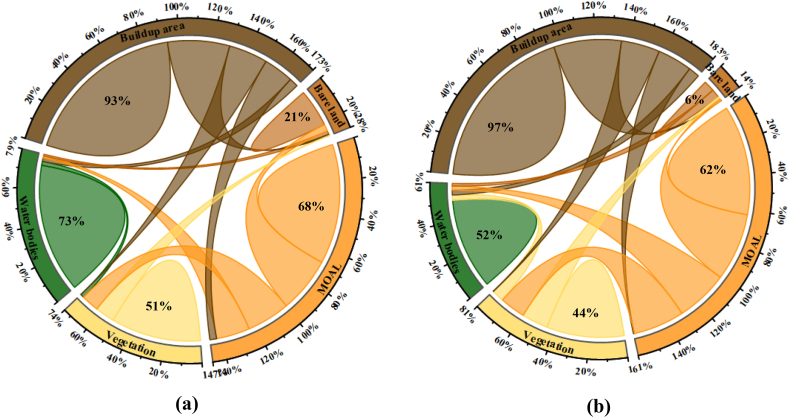
Markov chain transition probability of changing each Land use land cover class to another class (a) 2000–2010 (b) 2010–2020.

ANN-MLP could not show the future land demand for the growing buildup area. Markovian transition probability matrix had used in this regard to generate a map of each Land use likelihood of being that particular and generates a matrix to represent the probability of changing one class to another class within a specific time. Fig. 7(a) reveals that buildup areas and water bodies were the most stable Land use class with probabilities of 93% and 73% between 2000–2010, respectively. The most dynamic classes like bare land and vegetation had likelihood of 21% and 51%, respectively whereas bare land had a 35% probability of being converted into a buildup area. In Fig. 7(b), the transition of buildup area shows same type of stability, however, water bodies, bare land, vegetation and MOAL were the most dynamic LULC classes. From 2010 to 2020, water bodies and bare land shows the likelihood of drastic changes as 52%, 6%, respectively, with fcomparing to the previous periods that exhibits the huge pressure on resources. Likewise, bare land had the probability of 39% to convert into urban and some were converted into MOAL. Vegetation land cover contributed 22% to forming buildup area.

#### 3.3.2 Land use prediction and area statistics of LULC types

**Fig. 8:**
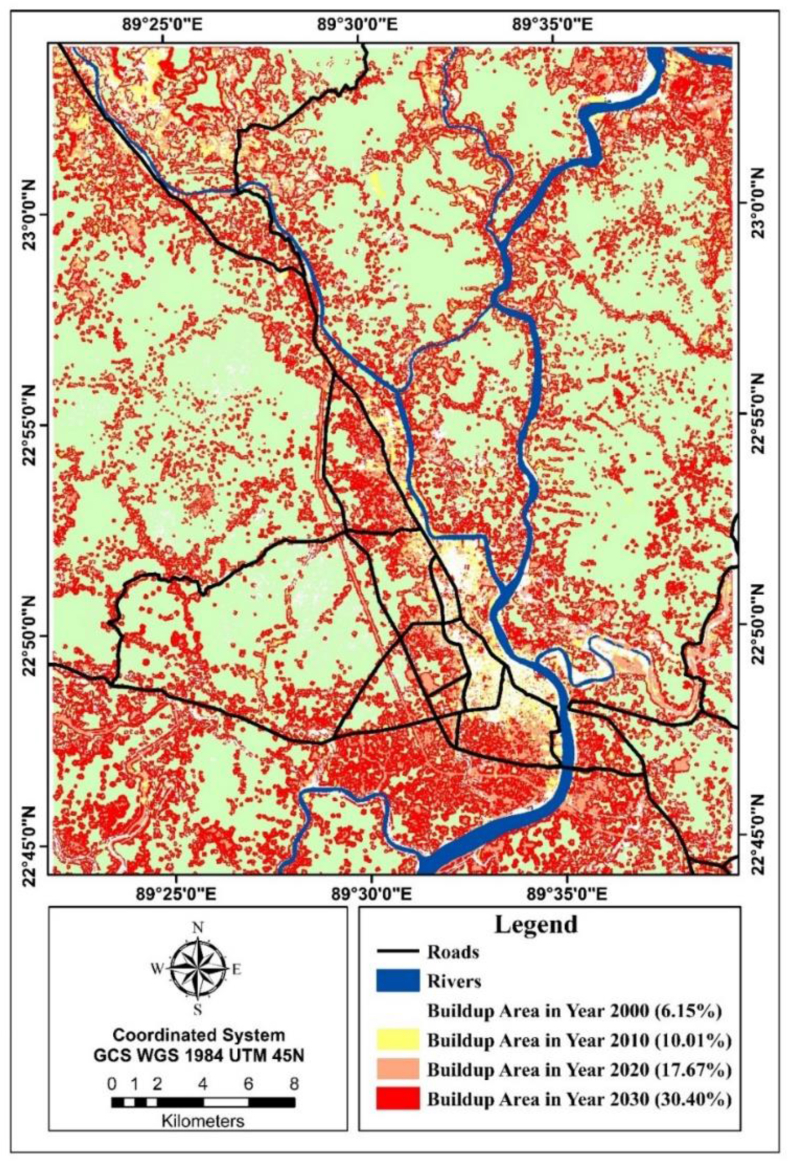
The actual urban area of 2000, 2010, 2020 and simulated urban area of 2030.

**Fig. 9:**
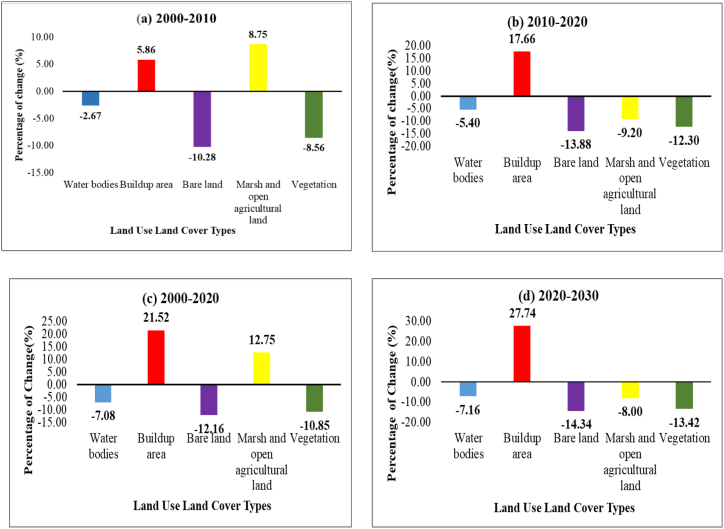
Gain and Loss of LULC Types for study area.

Table 8 shows that the simulated buildup area was recorded as 175 km^2^ whereas the actual buildup area was 194 km^2^ in 2020. It portrays the simulated map as slightly deviated from the actual scenario. Vegetation, MOAL were predicted with high accuracy. The area of simulated buildup area was 334 km^2^ by 2030 which is 30% of the total study area (Fig. 8). Fig. 9(a–d) illustrates that bare land reduced immensely by 12% in between 20 years period and was predicted to reduce by 14% by 2030 whereas 21% of buildup area was generated between 2000 and 2020. Therefore, buildup area demand created pressure on natural resources over the years.

**Table 8:** Prediction accuracy calculation between actual and simulated LULC map of 2020.

Land use land cover types	Actual 2020 LULC	Predicted 2020 LULC	Difference	Percentage	Prediction Accuracy (%)
1. Water bodies	27.68	32.98	−5.30	−19.15	80.85
2. Buildup area	194.32	175.83	18.49	9.52	90.48
3. Bare land	45.5	50.95	−5.45	−11.98	88.02
4. Marsh and open agricultural land (MOAL)	596.42	552.79	43.63	7.32	92.68
5. Vegetation	238.03	266.76	−28.73	−12.07	87.93

#### 3.3.3 Validation of urban growth simulation

**Fig. 10:**
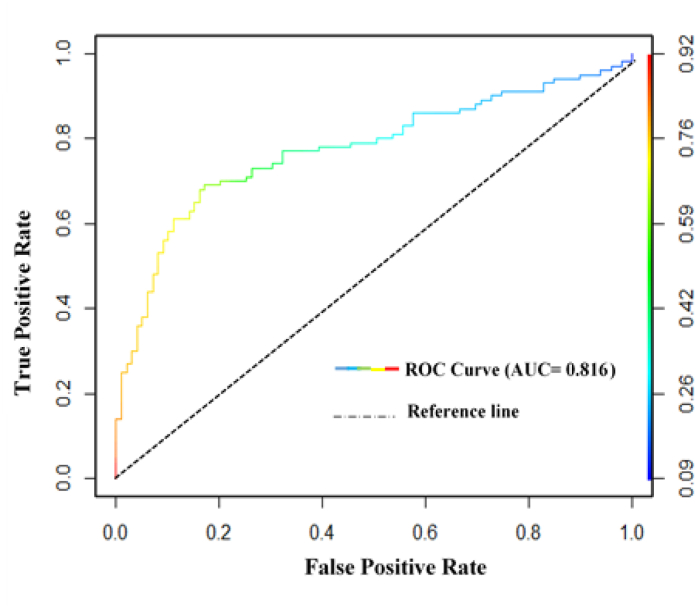
Relative operative characteristics (ROC) curve representing the correlation between simulated and actual map of 2020.

A detailed assessment was accomplished by using kappa and ROC curve (Relative Operating Characteristics). Kappa variations compared the simulated and actual Land use Land Cover map of 2020 with 87.09% of correctness, kappa (location) = 87.13, kappa (overall) = 85.30. Along with that, the ROC curve exibits the trade-off between the true positive rate and false-positive rate, where Area Under Curve (AUC) value was 0.816, that indicates the model has 82% power to simulate accurately LULC classes (Fig. 10). Here the value of kappa and ROC both indicates an acceptable consistency between simulated and actual Land use land cover scenario to forecast future urban expansion. As the research focused on simulating urban growth, the model is quite reliable for predicting future urban expansion to delineate UGB under different scenarios.

### 3.4 Delineation of Urban Growth Boundary

#### 3.4.1 Direction of urban spatial growth

**Fig. 11:**
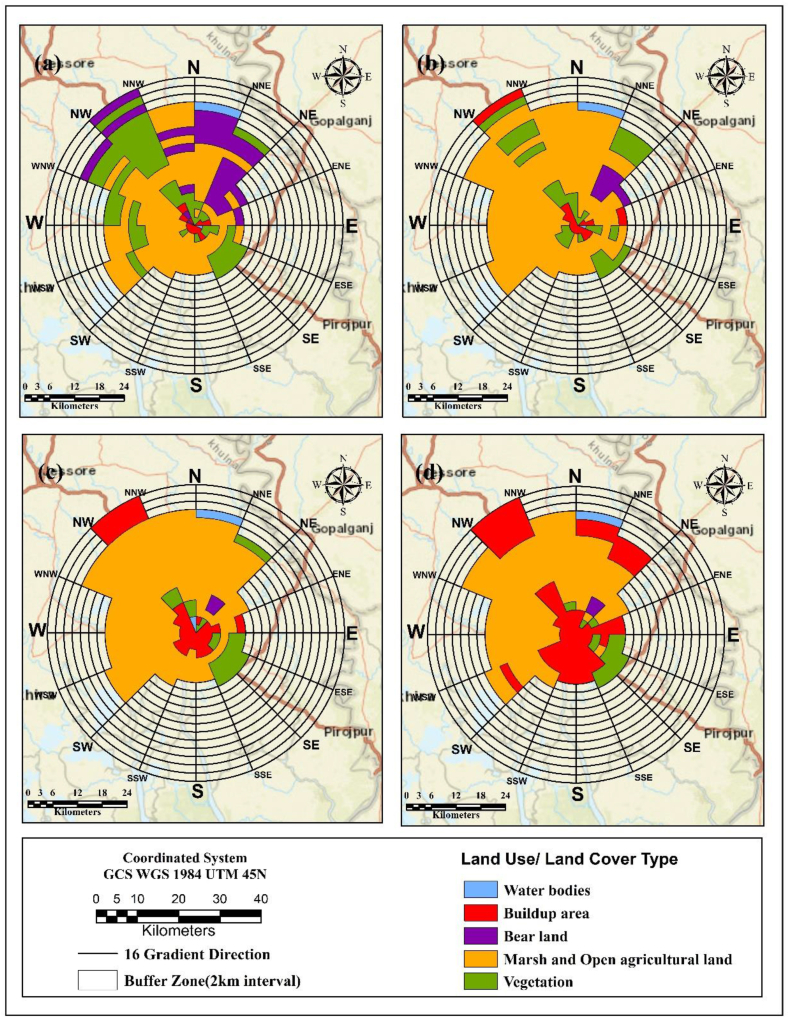
Gradient analysis of each Land use class in 16 gradient direction.

**Fig. 12:**
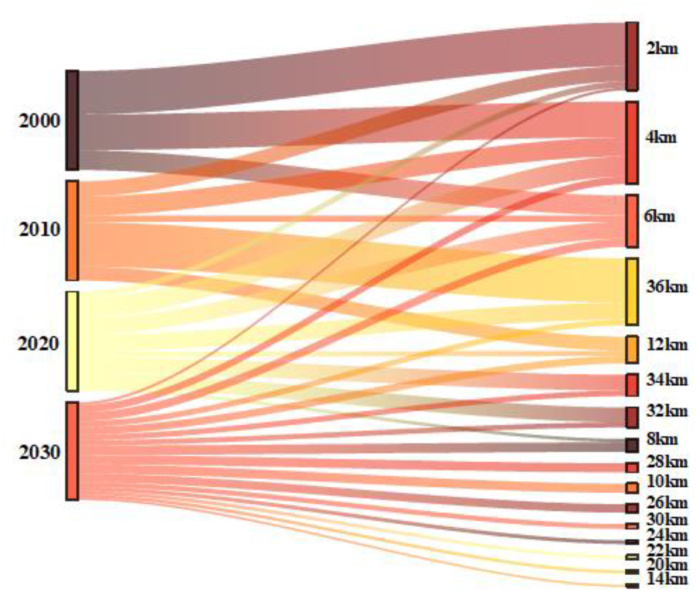
Amount of buildup area extends in 36km radius.

**Fig. 13:**
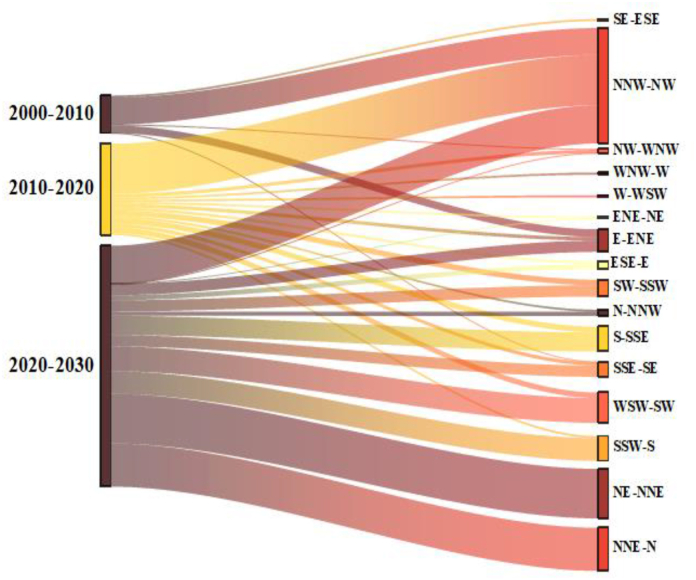
Amount of buildup area extends in 16 gradient direction.

Fig. 11 illustrates the historical trends and future direction of each Land use land cover pattern of the study area. 82% urban expansion had appeared within the city centre (KDA more) in 6 km radius, covering Kotawali thana towards Dighaliya upazila (Fig. 12, Fig. 13). Most of the urban growth is headed in the northwest direction whereas vegetation was replaced by a buildup area in the same direction. The southern part shows less growth and less conversion of vegetation cover comparatively.

#### 3.4.2 Spatial pattern quantification and urban landscape dynamics

**Fig. 14:**
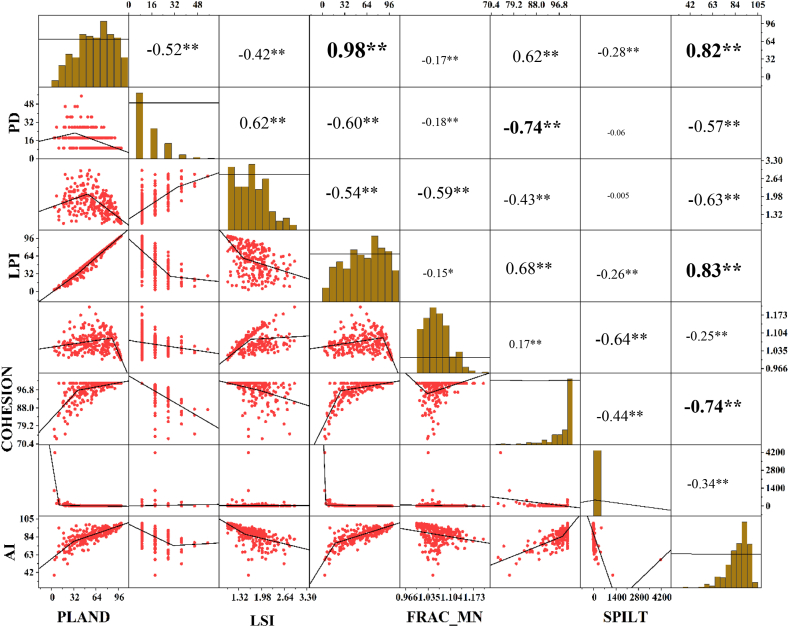
Correlation Scatter plot of selected landscape metrics.

Landscape metrics help to quantify the future pattern, shape, and perplexity of buildup area for local scale landscape. There have so many metrics that reveal different contexts to explain the pattern of urban growth. A correlation scatter plot was used to find out the relationship between the variables and the level of significance (Fig. 14). PLAND was positively correlated with LPI, COHESION, and AI and negatively correlated with PD, and LSI. However, PD negatively correlated with selected metrics except for LSI. This information helped to portray a comprehensive urban growth boundary.

#### 3.4.3 Landscape patterns of buildup growth

**Fig. 15:**
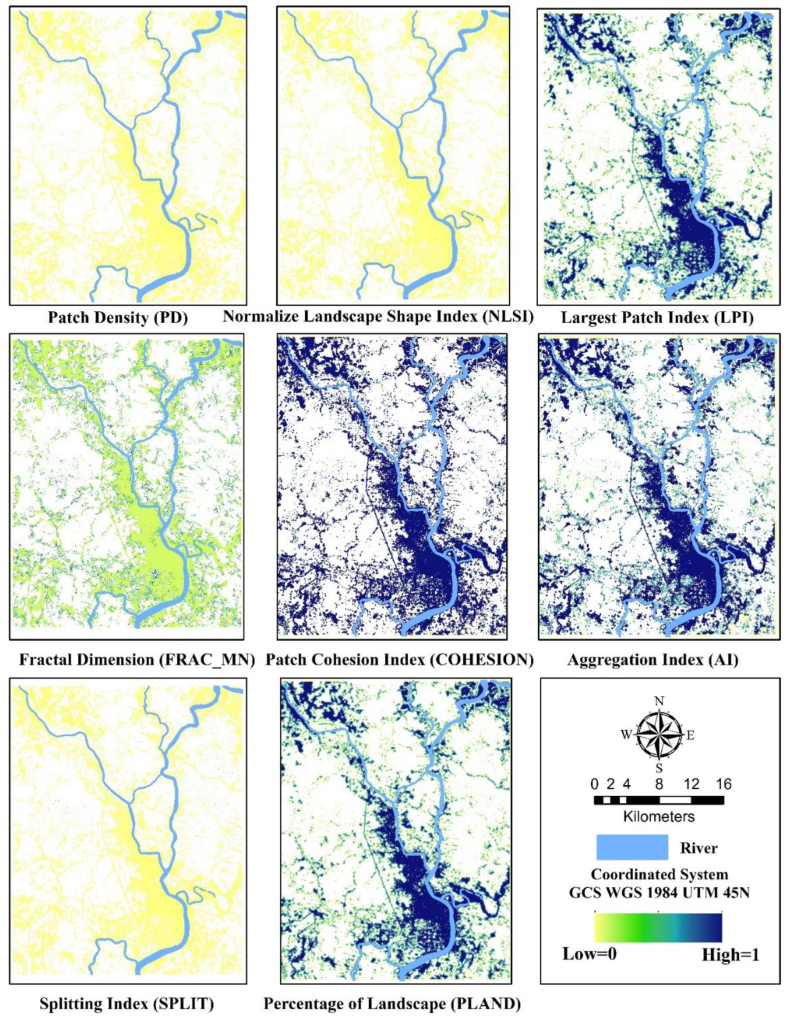
Selected landscape metrics.

**Fig. 16:**
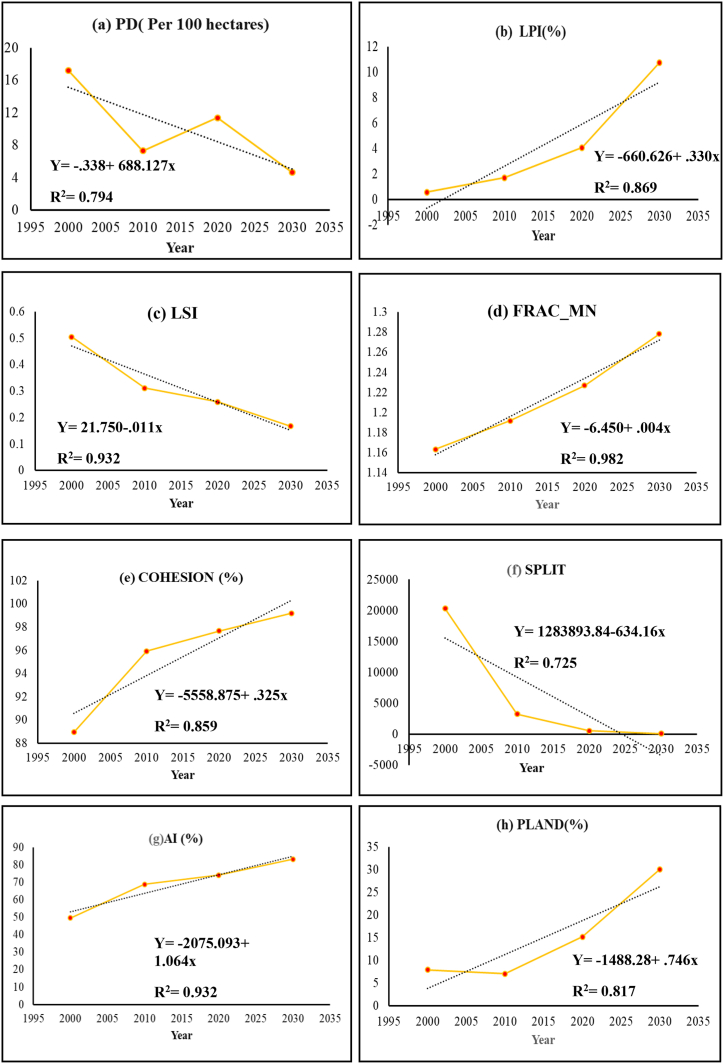
The landscape-level metrics of buildup area from 2000 to 2030.

The index value of PD (fragmentation index) ranging from (0–119.38) indicates a complex patch boundary at the interfaces of peri-urban areas outskirts of the KDA planning area (Fig. 15). LPI value (0–100) represents the area of the regional center that is predominant and highly connected. PLAND shows the same type of Characteristics. A low LSI value was found in the city center which indicated the patch aggregation. Moreover, LSI has decreased over the years indicating the increasing aggregation and decreasing complexity of patches (Fig. 16). The index value of FRAC_MN (0–1.25) shows the same type of unstructured sprawling pattern of urban growth. A high cohesion index value indicates the physical connection of the buildup area and how a particular patch type spreads and is dispersed. AI and COHESION have an inverse relation with the split index which shows less split in the urban area. The high value of LPI is detected in the potential and newly generated high-density urban area under the KDA Planning area whereas more aggregation is within the KCC area according to the Aggregation Index (AI) and fragmentation at the periphery of adjoining city boundary due to less administrative regulations. For managing growth and controlling land fragmentation, a suitable boundary may provide instrumental support.

**Fig. 17:**
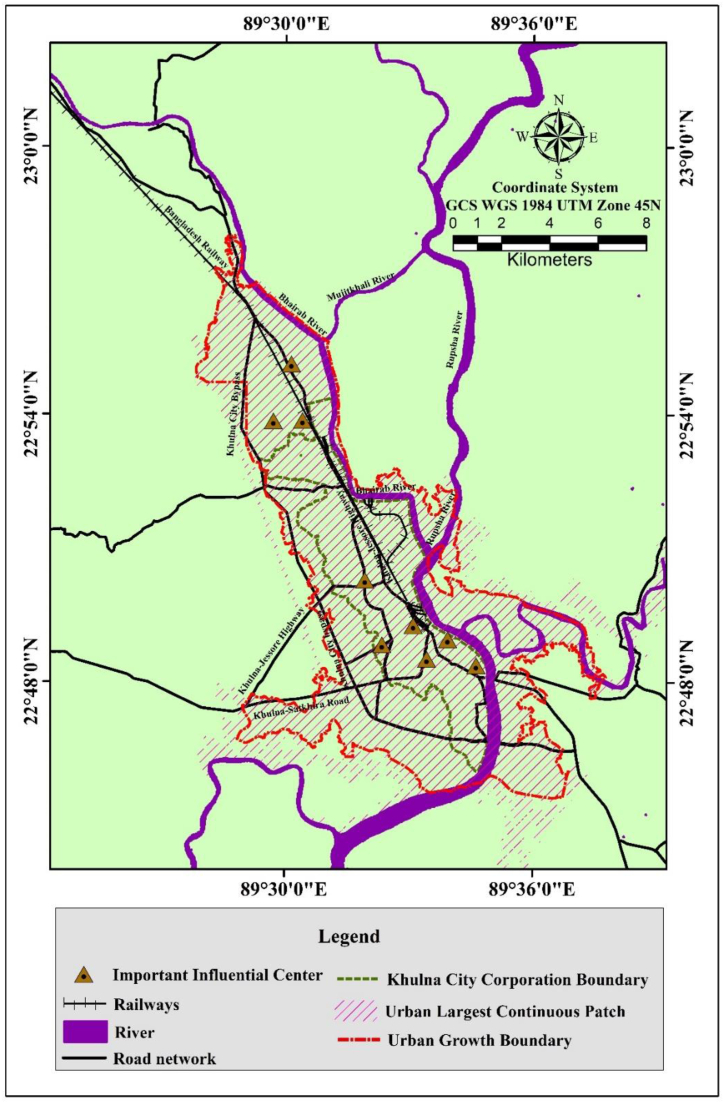
Delineation of urban growth boundary.

Nonetheless, one single metric is not able to portray the whole landscape scenario. PCA was used to address the variability of selected metrics and combined them into UCPI (ranges from 0.29 to 8.9). It explains 74% variance among the selected metrics. The compact patch (ranges from 2.00 to 3.00) was mostly found in the Khulna Metropolitan city area as well as Phultala Union (adjacent Growth center) which is located in the North-western direction. But the rest of the unions of Phultala upazila detached from the abundant patch and were excluded from UGB. Dumuria, Terokhada, and Batiaghata are identified as semi-compact suburb (>3.00). Incorporating CPI value, the Proposed UGB accounted for 140 km^2^ including (5 thana, 5 upazilas, 9 unions) Khulna Metropolitan area (55.70 km^2^), Phultala (Phultala, Damodarpur), Dumuria (Gutudia), Batiaghata (Jalma, Amirpur), Dighalia (Senhati), Rupsha (Aijganti, Naihati, Sreefaltala) (Fig. 17).

#### 3.4.4 Validation of contagious buildup area

According to the overarching objective of UGB is to encourage continuous urban growth, the contiguous buildup areas were simulated for 2020 in the TerrSet interface. On the other side, the actual urban continuous patch was determined by the landscape metrics for 2020. After that, the actual and predicted continuous buildup areas were compared by the kappa co-efficient with the accuracy of 83.61%.

#### 3.4.5 Service gap identification

**Fig. 18:**
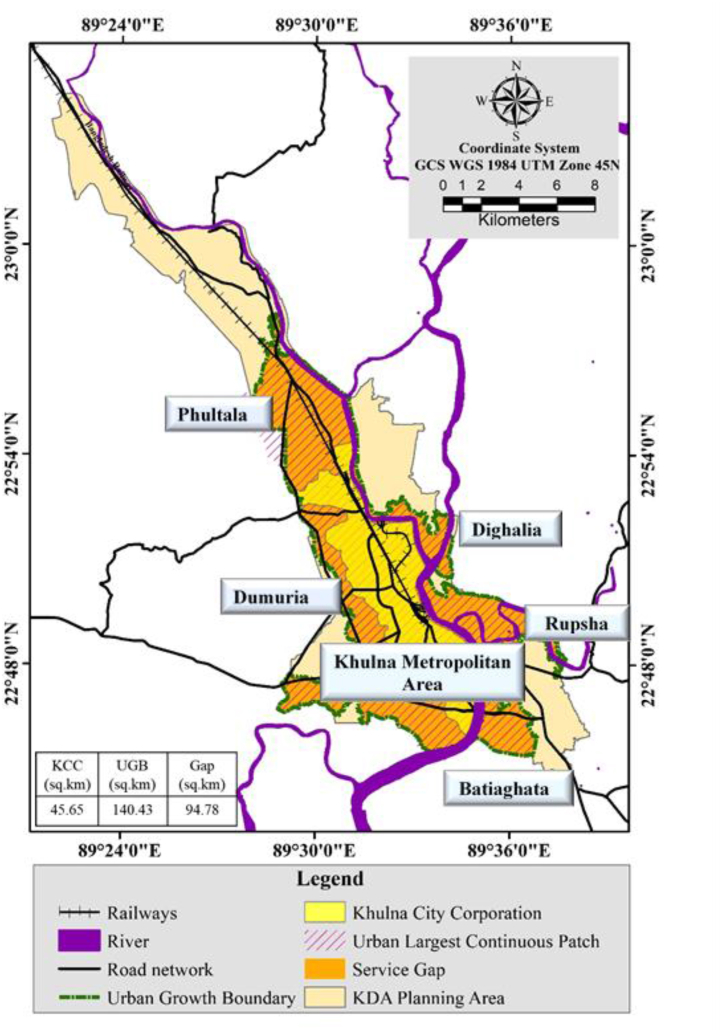
Comparison among urban growth boundary, KCC and KDA

(Fig. 18) shows that the UGB exceeds the boundary of the KCC area which portrays a visible gap (94.78 km^2^) between the supply and demand of service facilities. Improved sewage treatment and disposal, water purification and supply, roads and highways, recreation facilities, and solid waste management are just available under KCC jurisdiction. As the urban largest continuous patch will likely cover a huge area in the future, the facilities and amenities will be required for increased urban areas. KDA has the power to control future urban growth by demarcating UGB and can influence KCC to increase its service under the growth boundary.

## 4 Discussion

In this research, we propose a framework of an urban growth model with significant explanatory variables to demarcate an UGB for Khulna city. The main goal is to quantify urban landscape dynamics and delineate an UGB using spatial analysis and landscape metrics for 2030.

Several studies had applied UGB in their studies outside Bangladesh [[22], [24], [25], [60], [61], [62]]; in India, China, Iran with effective way. By comparing these studies with ours, this research has some unique features that promote feasible urban management in Khulna city: For Shiliguri, India, urban growth models did not count the biophysical factors; for example, elevation and slope are the most dominating factor for shaping the city (Stucture Plan, Khulna City, 2002). In the study of Kolkata, India and Wuhan, China, land price was not accounted for delineating UGB and lack of directional specification over the years. Moreover, the gap between the existing supply and demand of services was not accounted for in the previous research while demarcating a boundary.

In developing countries, ‘Urban Service Area’ cannot control urban growth alone and provide service facilities inside the jurisdiction of KCC, Nevertheless, KDA has the power for controlling the development and land use under its boundary. Planning authorities and decision-makers are dealing with how much land will get transformed into an urban area and the direction where land conversion will be extended in future. Therefore, shape and direction of future growth of buildup area help them for effective management of the urban area as well as natural resources of Khulna city. However, Khulna city master plan is the sole document for planning and development for controlling infrastructure development. This study illustrated how significant the delineated UGB is to add in the upcoming master plan of Khulna City.

This research used an Artificial Neural Network (ANN) based approach to predict future urban growth. For model calibration and validation, we used multi-temporal LULC maps of 2000, 2010, 2020. The whole study followed two validation methods of urban growth simulation. The overall accuracy of kappa was 85.30% and AUC is 0.816 which presented high accuracy of the model and a slight difference between actual and simulated urban growth.

Urban growth model and local information on urban patches both are essential to formulate a comprehensive plan [[14], [17], [25]]. ignore the urban patch information and did not incorporate the information about how much the newly generated urban patch will increase in future at a local level. According to our study, a Compact patch was mostly found under the Khulna Metropolitan area whereas fragmented and unstructured patch were recorded between urban-rural interfaces. Patch information helps to set development goals, and policies and reduce the adverse consequences of urban growth. In Bangladesh, there have several loopholes in planning schemes, hence, a comprehensive and flexible UGB can resolve this issue by taking location-specific parameters.

Khulna's landscape experienced a significant shift between 2000 and 2020, with a 12% and 10% decrease in bare land and vegetation, respectively, by an increasing 21% of developed land. This research quantifies the rate of urban expansion as well as other land use changes in several directions. The most dominant urban expansion was noticed in the northwest direction over the years. The construction Khulna-Jessor road shapes the Khulna city towards the north-western direction and the Noapara bypass road adjoining it on the north. Fast urban growth was also observed due to suitable land elevation (4 ft to 5 ft.) for urban settlement and road accessibility on the west; for example, the establishment of Khulna University, Khulna city bypass road and Khulna-Satkhira road[38]. This trend was predicted to continue further by 2030. Nevertheless, the southnorth ranks slower growth than the north. After the inclusion of the eastern side of Rupsha and Dighalia thanas in the master plan, shrimp processing firms lead to physical growth in the south-eastern direction. Without proper development regulation, it would have strong possibility to the expansion of the city towards the southeast and northwest direction, where natural resources wil be converted into urban areas. As UGB exceeds the boundary of the KCC area, KCC should extend its service area to balance the demand of continuous urban growth. And KDA needs to restrict urban growth in UGB areas to minimize the cost of service facilities.

Astonishingly, Bangladesh has yet to have any comprehensive regulatory framework to solve the impact of urban sprawl. In recent times, KDA introduce Detail Area Plan (DAP) into the planning stream but could not specifically work on growth control measures. Prediction failure, lack of proper effective land use policies and legal measures, and lack of proper land distribution are the main barrier to upholding the provision of the master plan and controlling urban expansion. Bare land and vegetation are transformed into open agricultural land and then converted into buildup areas, which encourages sprawling in peri-urban areas. A flexible approach would be advantageous in proper land management and distribution mechanism. Implementation of UGB helps to ensure optimal use of land and manage urban-rural interface. There has around 95 km^2^ gap between the existing service provided by KCC and the future demand of Khulna city. The effective implication of UGB by KDA helps to control urban continuous growth.

## 5 Conclusions

Khulna's Landscape has experienced tremendous changes between 2000 and 2020 and this trend will possibly continue by 2030, influenced by its impetuous economic growth and infrastructure development. For planners, proper modelling of UGB acts as a reference which enables to determine the trajectory of urban growth. In this research, an integrated structure was introduced to delineate a UGB for a metropolitan city of Bangladesh. An artificial neutral Network-based approach is applied to project an urban hard boundary depending upon some explanatory variables and landscape metrics. The following important conclusion can be drawn:

(1) Based on urban growth simulation, the urban area increased from 194 km^2^ in 2020 to 334 km^2^ in 2030. It indicates that Khulna's urbanization will continue rapidly in future without necessary development control schemes. Furthermore, due to increased economic growth, the urban growth of Khulna impacts the existing urban areas and fringe areas by creating locational advantages.
(2) Simulation result shows that the intensity of urban growth will rise in the North-western direction having good road connectivity and comparatively low urban footprint in the southeast direction.
(3) Gap identification of existing supply and demand depicts KCC will not meet up the future demand of urban continuous patch area. As UGB is under KDA jurisdiction, it has the power to control this continuous growth inside its jurisdiction.
(4) This study could not include all explanatory variables due to lack of available income and employment data. The government and concerned authorities should come forward to manage those data which helps for precise gap identification and flexible UGB demarcation.

This research will effectively guide the future urban expansion while mobilizing the services and utilities by reducing the environmental cost of infrastructures and developments in the region. A number of potential future road maps would be deemed for efficient implementation of policy for Khulna. Along with simulating future urban growth direction, further investigation on the socio-economic datasets, such as, economic activities of the residents, slum settlement and their role as a part of UGB demarcation and informal settlements of Khulna city would need to be considered to make it a holistic approach. Additionally, there are some unforeseen factors, like political pressure and control of private developers made it difficult to run the model successfully, although, simulation outcome come up with good approximation. Hence, frequent monitoring and evaluation would need to adjust the UGB delineation regarding the changes of any factor for maintaining sustainable urban growth in Khulna.

## Author contribution statement

Arpita Bakshi, BURP: Conceived and designed the experiments; Performed the experiments; Analyzed and interpreted the data; Wrote the paper. Md. Esraz-Ul-Zannat: Analyzed and interpreted the data; Contributed reagents, materials, analysis tools or data; Wrote the paper.

## Data availability statement

Data will be made available on request.

## Declaration of competing interest

The authors declare that they have no known conflict of interest of financial or personal interest and belief that could affect the work presented in this study. This work is funded by the authors themselves. All authors have reviewed and approved the final manuscript.

## References

[1] United Nations (2015).

[2] UNDESA (2018). https://population.un.org/wup/Publications/Files/WUP2018-Report.pdf.

[3] Planning Commission. (2012). Perspective Plan of Bangladesh 2010-2021: Making Vision 2021 a reality. Government of the People’s Republic of Bangladesh, April, 110, http://bangladesh.gov.bd/sites/default/files/files/bangladesh.gov.bd/page/6dca6a2a_9857_4656_bce6_139584b7f160/Perspective-Plan-of-Bangladesh.pdf.

[4] S. Roy, T. Sowgat, M. Uddin, A. S. M. T. Islam, N. Anjum, J. Mondal & M. M. Rahman, (2018), Bangladesh: National Urban Policies and City Profiles for Dhaka and Khulna. January, 156. http://www.centreforsustainablecities.ac.uk/wp-content/uploads/2018/06/Research-Report-Bangladesh-National-Urban-Policies-and-City-Profiles-for-Dhaka-and-Khulna.pdf.

[5] Han H., Yang C., Song J. (2015). Scenario simulation and the prediction of land use and land cover change in Beijing, China. Sustainability.

[6] (2015). Planning Commission, “7th Five Year Plan (FY2016-FY2020): Accelerating Growth, Empowering Citizens,” in Government of the People’S Republic of Bangl. General Economic Division.

[7] Sinclair-smith K. (2013).

[8] Tannier C., Thomas I., Vuidel G., Frankhauser P. (2011). A fractal approach to identifying urban boundaries. Geogr. Anal..

[9] Baqa M.F., Chen F., Lu L., Qureshi S., Tariq A., Wang S., Jing L., Hamza S., Li Q. (2021). Monitoring and modeling the patterns and trends of urban growth using urban sprawl matrix and CA-Markov model: A case study of Karachi, Pakistan. Land.

[10] Falah N., Karimi A., Harandi A.T. (2020). Urban growth modeling using cellular automata model and AHP (case study: Qazvin city). Modeling Earth Systems and Environment.

[11] Jat M.K., Choudhary M., Saxena A. (2017). Application of geo-spatial techniques and cellular automata for modelling urban growth of a heterogeneous urban fringe. Egyptian Journal of Remote Sensing and Space Science.

[12] Bibri, S. E., Krogstie, J., & Kärrholm, M. (2020). Compact city planning and development: Emerging practices and strategies for achieving the goals of sustainability. *Developments in the Built Environment*, *4*(June). 10.1016/j.dibe.2020.100021.

[13] Ewing R., Cervero R. (2017). Does Compact Development Make People Drive Less?” The Answer Is Yes. Journal of the American Planning Association.

[14] Bhatta B. (2009). Modeling of urban growth boundary using geoinformatics. Int. J. Digit. Earth.

[15] Jiang P., Cheng Q., Gong Y., Wang L., Zhang Y., Cheng L., Li M., Lu J., Duan Y., Huang Q., Jiang P., Cheng Q., Gong Y., Wang L., Zhang Y., Cheng L. (2016). Using urban development boundaries to constrain uncontrolled urban sprawl in China using urban development boundaries to constrain uncontrolled urban sprawl in China.

[16] Mathur S. (2019). Impact of an urban growth boundary across the entire house price spectrum: the two-stage quantile spatial regression approach. Land Use Pol..

[17] Tayyebi A., Perry P.C., Tayyebi A.H. (2014).

[18] Ma S., Li X., Cai Y. (2017). Delimiting the urban growth boundaries with a modified ant colony optimization model. Comput. Environ. Urban Syst..

[19] Hashemi Aslani Z., Omidvar B., Karbassi A. (2022). Integrated model for land-use transformation analysis based on multi-layer perception neural network and agent-based model. Environmental Science and Pollution Research.

[20] Hishe S., Bewket W., Nyssen J., Lyimo J. (2020). Analysing past land use land cover change and CA-Markov-based future modelling in the Middle Suluh Valley, Northern Ethiopia. Geocarto Int..

[21] Yi S., Zhou Y., Li Q. (2022). A New Perspective for Urban Development Boundary Delineation Based on the MCR Model and CA-Markov Model. Land.

[22] Chakraborti S., Das D.N., Mondal B., Shafizadeh-Moghadam H., Feng Y. (2018). A neural network and landscape metrics to propose a flexible urban growth boundary: a case study. Ecol. Indicat..

[23] Gharaibeh A.A., Jaradat M.A., Kanaan L.M. (2023). A Machine Learning Framework for Assessing Urban Growth of Cities and Suitability Analysis. Land.

[24] Zhang D., Liu X., Lin Z., Zhang X., Zhang H. (2020). The delineation of urban growth boundaries in complex ecological environment areas by using cellular automata and a dual-environmental evaluation. J. Clean. Prod..

[25] He Q., Tan R., Gao Y., Zhang M., Xie P., Liu Y. (2018). Modeling urban growth boundary based on the evaluation of the extension potential: a case study of Wuhan city in China. Habitat Int..

[26] Tayyebi A., Perry P.C., Tayyebi A.H. (October 2014).

[27] Taubenbock H., Wegmann M., Roth A., Mehl H., Dech S. (2009). Computers , environment and urban Systems urbanization in India – spatiotemporal analysis using remote sensing data. Comput. Environ. Urban Syst..

[28] Sowgat T. (2012).

[29] Khulna Developemnt Authority. (2000). *Structure Plan, Master Plan and Detailed Area Plan for Khulna City. *Volume II: Structure Plan* (July 2002).*

[30] Bangladesh Bureau of Statistics (Bbs) (2013).

[31] Khulna Development Authority (2000). http://www.tno.nl/content.cfm?context=thema&conten.

[32] Feng Y., Liu Y., Tong X. (2018). Spatiotemporal variation of landscape patterns and their spatial determinants in Shanghai, China. Ecol. Indicat..

[33] Szilassi P., Bata T., Szabó S., Czucz B., Molnar Z., Mezosi G. (2017). The link between landscape pattern and vegetation naturalness on a regional scale. Ecol. Indicat..

[34] Ball M., Taylor E., Wood G. (2014). Urban growth boundaries and their impact on land prices.

[35] BBS (2015).

[36] Bierwagen B.G. (2005). Predicting ecological connectivity in urbanizing landscapes.

[37] Cho S.-H., Omitaomu O.A., Poudyal N.C., Eastwood D.B. (2007). The impact of an urban growth boundary on land development in Knox county, Tennessee: a comparison of two-stage probit least squares and multilayer neural network models. J. Agric. Appl. Econ..

[38] Khulna Developemnt Authority (2000).

[39] Guest A.M. (1973). Urban growth and population densities.

[40] Guneralp B., Reba M., Hales B.U., Wentz E.A., Seto K.C. (2020).

[41] Congalton R.G. (1991). A review of assessing the accuracy of classifications of remotely sensed data. Rem. Sens. Environ..

[42] Mozumder C., Tripathi N.K. (2014). Geospatial scenario based modelling of urban and agricultural intrusions in Ramsar wetland deepor beel in northeast India using a multi-layer perceptron neural network. Int. J. Appl. Earth Obs. Geoinf..

[43] Rumelhart D.E., Hinton G.E., Williams R.J. (1986). Learning representations by back-propagating errors. Nature.

[44] Dadhich P.N., Hanaoka S. (2010). Markov method integration with multi-layer perceptron classifier for simulation of urban growth of Jaipur city. International Conference on Electric Power Systems, High Voltages, Electric Machines, International Conference on Remote Sensing - Proceedings.

[45] Geneletti D. (2013). Assessing the impact of alternative land-use zoning policies on future ecosystem services. Environ. Impact Assess. Rev..

[46] Eastman J.R. (2012). IDRISI Selva Tutorial. Idrisi Production, Clark Labs-Clark University.

[47] Eastman J.R. (2020). TerrSet 2020: Geospatial Monitoring and Modeling System. Clark Labs.

[48] Mandrekar J.N. (2010). Receiver operating characteristic curve in diagnostic test assessment. J. Thorac. Oncol..

[49] Qiao Z., Tian G., Zhang L., Xu X. (2014). Influences of urban expansion on urban heat island in Beijing during 1989-2010. Adv. Meteorol..

[50] Bian F., Xie Y., Cui X., Yz (2014). Geo-Informatics in Resource Management and Sustainable Ecosystem. In International Archives of the Photogrammetry, Remote Sensing and Spatial Information Sciences - ISPRS Archives.

[51] Al-Sharif A.A.A., Pradhan B., Shafri H.Z.M., Mansor S. (2014). Quantitative analysis of urban sprawl in Tripoli using Pearson’s Chi-Square statistics and urban expansion intensity index. IOP Conf. Ser. Earth Environ. Sci..

[52] Shi G., Jiang N., Li Y., He B. (2018). Analysis of the dynamic urban expansion based on multi-sourced data from 1998 to 2013: a case study of Jiangsu Province. Sustainability.

[53] Nichol J.E., Abbas S., Fischer G.A. (2017). Spatial patterns of degraded tropical forest and biodiversity restoration over 70-years of succession. Glob. Ecol. Conserv..

[54] Liu S., Zhang X., Feng Y., Xie H., Jiang L., Lei Z. (2021). Spatiotemporal dynamics of urban green space influenced by rapid urbanization and land use policies in Shanghai. Forests.

[55] Hasan S., Shi W., Zhu X., Abbas S., Khan H.U.A. (2020). Future simulation of land use changes in rapidly urbanizing South China based on land change modeler and remote sensing data. Sustainability.

[56] McGarigal K., Marks B.J. (1995).

[57] Allen M.P. (2007). Understanding Regression Analysis.

[58] Cushman S.A., McGarigal K., Neel M.C. (2008). Parsimony in landscape metrics: strength, universality, and consistency. Ecol. Indicat..

[59] Gwelo A.S. (2019). Principal components to overcome multicollinearity problem. Oradea J. Bus. Econom..

[60] Liang X., Liu X., Li X., Chen Y., Tian H., Yao Y. (2018). Delineating multi-scenario urban growth boundaries with a CA-based FLUS model and morphological method. Landsc. Urban Plann..

[61] Tayyebi A., Pijanowski B.C., Pekin B. (2011). Two rule-based urban growth boundary models applied to the tehran metropolitan area, Iran. Appl. Geogr..

[62] Khulna Developemnt Authority (2000).

